# Carbon Amendments Alter Microbial Community Structure and Net Mercury Methylation Potential in Sediments

**DOI:** 10.1128/AEM.01049-17

**Published:** 2018-01-17

**Authors:** Geoff A. Christensen, Anil C. Somenahally, James G. Moberly, Carrie M. Miller, Andrew J. King, Cynthia C. Gilmour, Steven D. Brown, Mircea Podar, Craig C. Brandt, Scott C. Brooks, Anthony V. Palumbo, Judy D. Wall, Dwayne A. Elias

**Affiliations:** a Biosciences Division, Oak Ridge National Laboratory, Oak Ridge, Tennessee, USA; b Department of Soil and Crop Sciences, Texas A&M University, Overton, Texas, USA; c Department of Chemical and Materials Engineering, University of Idaho, Moscow, Idaho, USA; d Department of Biology, Troy University, Troy, Alabama, USA; e Smithsonian Environmental Research Center, Edgewater, Maryland, USA; f Environmental Sciences Division, Oak Ridge National Laboratory, Oak Ridge, Tennessee, USA; g Department of Biochemistry, University of Missouri, Columbia, Missouri, USA; Chinese Academy of Sciences

**Keywords:** methylmercury, 16S, qPCR, *hgcA*, *hgcAB*, mercury

## Abstract

Neurotoxic methylmercury (MeHg) is produced by anaerobic Bacteria and Archaea possessing the genes *hgcAB*, but it is unknown how organic substrate and electron acceptor availability impacts the distribution and abundance of these organisms. We evaluated the impact of organic substrate amendments on mercury (Hg) methylation rates, microbial community structure, and the distribution of *hgcAB^+^* microbes with sediments. Sediment slurries were amended with short-chain fatty acids, alcohols, or a polysaccharide. Minimal increases in MeHg were observed following lactate, ethanol, and methanol amendments, while a significant decrease (∼70%) was observed with cellobiose incubations. Postincubation, microbial diversity was assessed via 16S rRNA amplicon sequencing. The presence of *hgcAB^+^* organisms was assessed with a broad-range degenerate PCR primer set for both genes, while the presence of microbes in each of the three dominant clades of methylators (Deltaproteobacteria, Firmicutes, and methanogenic Archaea) was measured with clade-specific degenerate *hgcA* quantitative PCR (qPCR) primer sets. The predominant microorganisms in unamended sediments consisted of Proteobacteria, Firmicutes, Bacteroidetes, and Actinobacteria. Clade-specific qPCR identified *hgcA^+^* Deltaproteobacteria and Archaea in all sites but failed to detect *hgcA*^+^ Firmicutes. Cellobiose shifted the communities in all samples to ∼90% non-*hgcAB*-containing Firmicutes (mainly Bacillus spp. and Clostridium spp.). These results suggest that either expression of *hgcAB* is downregulated or, more likely given the lack of 16S rRNA gene presence after cellobiose incubation, Hg-methylating organisms are largely outcompeted by cellobiose degraders or degradation products of cellobiose. These results represent a step toward understanding and exploring simple methodologies for controlling MeHg production in the environment.

**IMPORTANCE** Methylmercury (MeHg) is a neurotoxin produced by microorganisms that bioacummulates in the food web and poses a serious health risk to humans. Currently, the impact that organic substrate or electron acceptor availability has on the mercury (Hg)-methylating microorganisms is unclear. To study this, we set up microcosm experiments exposed to different organic substrates and electron acceptors and assayed for Hg methylation rates, for microbial community structure, and for distribution of Hg methylators. The sediment and groundwater was collected from East Fork Poplar Creek in Oak Ridge, TN. Amendment with cellobiose (a lignocellulosic degradation by-product) led to a drastic decrease in the Hg methylation rate compared to that in an unamended control, with an associated shift in the microbial community to mostly nonmethylating Firmicutes. This, along with previous Hg-methylating microorganism identification methods, will be important for identifying strategies to control MeHg production and inform future remediation strategies.

## INTRODUCTION

In nature, methylmercury (MeHg) production is predominately microbial (1) and limited to anaerobic microorganisms (2, 3). Mercury (Hg) methylation has been linked to reduction of sulfate (4–6) and Fe(III) (7, 8) as well as methanogenesis (9, 10), but only a subset of microorganisms are capable of methylating Hg within these functional groups. Three major Hg-methylating clades of microbes have been defined (11), including the Deltaproteobacteria, encompassing sulfate- and iron(III)-reducing bacteria, the Gram-positive Firmicutes, including sulfate- and sulfite-reducing bacteria, dehalogenators, and syntrophs, and methanogens within the class *Methanomicrobia*. The Deltaproteobacteria have been shown to be the predominant Hg methylators and display the highest Hg methylation efficiencies, while several Firmicutes and Archaea also have this function (2, 3).

Both inorganic Hg and MeHg are commonly observed in environments such as river sediments (12). MeHg is typically a small proportion of the total Hg (THg) in water and sediments; however, due to its toxicity and ability to biomagnify in food webs (13), MeHg poses a significant threat to ecosystems and human health. To date, Hg methylation has been characterized only for anaerobic microorganisms (2) possessing the gene pair *hgcAB* (3). As a result, Hg-contaminated bottom sediments with anaerobic conditions can be a significant source for MeHg production in water bodies. While several studies have investigated the geochemical controls on Hg methylation and the MeHg production potential (MPP), including salinity, pH, dissolved O_2_, the presence of sulfate and Fe(III), and alternate electron acceptors (7, 14–23), we are not aware of any study investigating the effect of specific carbon (C) compounds on MPP and the distribution of different types of methylating microbes in environmental samples. Further, while *hgcA* was originally annotated as a carbon monoxide dehydrogenase in Desulfovibrio desulfuricans ND132 (24) or a conserved hypothetical protein in Geobacter sulfurreducens PCA (25), the native function of HgcAB has to date not been explicitly determined. Hence, it is unknown how available carbon sources may affect MPP and *hgcAB*^+^ microbial diversity. Consequently, this gap in knowledge limits our ability to understand and predict what, if any, changes in MPP may occur as a consequence of anaerobic environments receiving complex carbon substrates and consistently high levels of atmospheric Hg, which can become a direct source of human exposure to MeHg (26).

To study the role that complex carbon sources may have in the MPP, we utilized East Fork Poplar Creek (EFPC) near Oak Ridge, TN, which is contaminated with Hg from the nearby Y-12 facility (see Fig. S1 in the supplemental material) (12, 27–30). Two sites along EFPC and one from the uncontaminated Hinds Creek (HC) were selected, each with different THg and MeHg concentrations (31–33). In general, sediment THg concentrations in bulk traditionally ranged from 10 to 50 mg/kg (dry weight) along the river bottom (12). MeHg was not released from the Y-12 facility, but THg was (12), and the MeHg found downstream is assumed to be generated within the creek. Separate incubations were set up *in vitro* with sediment from the three locations, and each was amended with one of six complex carbon sources to determine the MPP (via ^201^Hg spiking experiments) (34), alterations to the microbial community structure (via 16S rRNA gene sequencing) (35), and *hgcA* genome copy number (via quantitative PCR [qPCR]) (11). The carbon amendments (cellobiose, lactate, ethanol, methanol, acetate, and propionate) were included to mimic microbial exposure to common intermediates within the carbon cycle and to assess the impact or role each intermediate has in the diversity and abundance of microbial community populations and net Hg methylation production.

For this study we had several objectives in mind. First, we wanted to confirm the depth at which MeHg production (or MPP) was greatest for the particular samples studied, which we hypothesized to be at the lower anaerobic depths. Second, we wanted to determine which, if any, carbon compounds had an effect on MeHg production and microbial diversity. We hypothesized that the addition of cellobiose, a lignocellulose breakdown product, would stimulate Firmicutes specifically, since they are well known to degrade lignocellulose (36–38). Third, we wanted to compare 16S rRNA gene sequence counts of known Hg methylators to our recently developed qPCR-based *hgcA* protocol for determining Hg-methylating microorganism abundance. We determined that MeHg concentrations do not correlate well to either method in the context of the small sample size used in this study.

## RESULTS AND DISCUSSION

### Hg, MeHg, and MPP in natural sediments.

Sediment THg concentrations at the two EFPC sites ranged from 0.5 to 5.5 μg/g, with higher concentrations in deeper sediments and at the downstream NH site compared to the NOAA site (see Table S1 in the supplemental material). Ambient MeHg concentrations were also generally higher in deeper sediments and higher at the EFPC site further downstream (NH) (400 to 1200 pg/g) than at NOAA (170 to 550 pg/g), consistent with previous studies (31). The background site, HC, had THg and MeHg concentrations that were approximately 100 to 1,000 times less than those at the NOAA or NH site. In all cases, the percentage of THg as MeHg was low (0.002 to 0.010%), which is typical of Hg-contaminated sites, likely because Hg-methylating microbes are a small percentage of the total microbial community (2, 3, 39).

Mercury methylation potentials (measured as the fraction of an Hg spike methylated in 24 h) were similar across the sites and were similar or increased with depth (Table S1). Like the percentage of MeHg in these sediments, methylation rates in stream bottom sediments were low, on the order of 0.1 × 10^−4^ to 1.0 × 10^−4^ per day. Potential methylation rates in fine-grained organic sediments or saturated marsh soil, measured with Hg spikes following similar methods, tend to average ∼1,000 times more (0.1 × 10^−1^ to 1.0 × 10^−1^) (20). MPP and MeHg concentrations were used to select sediment depth for the more detailed experiments that addressed the effect of C compound on MPP at a single depth across all sites. The MPPs in the NOAA samples were similar with depth, with overlapping standard deviations, while the highest MPP was observed in the 8- to 12-cm depth interval from the NH site. Hence, the 8- to 12-cm interval sediments were used for subsequent experiments.

### Hg methylation response to carbon amendments.

**FIG 1:**
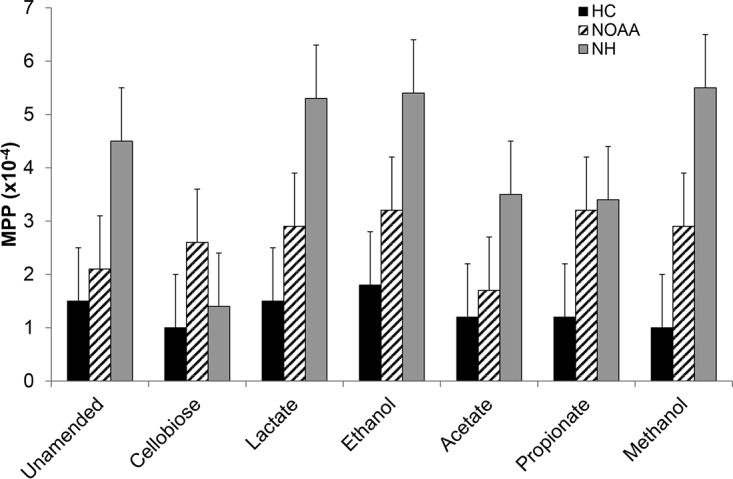
Methylmercury production potential (MPP) in microcosm experiments from each of three sites receiving one of seven carbon amendment treatments. MPP is the amount of MeHg produced per day (micrograms of MeHg per kilogram of THg spiked per day) or the fraction methylated (MeHg produced per THg added per day). Bars and error bars represent the mean ± standard deviation from triplicate microcosms. HC, Hinds creek (background site); NOAA, upstream site in the contaminated EFPC; NH, downstream site in EFPC.

Of the various organic substrate amendments made to the HC, NOAA, and NH slurries, only the polysaccharide (cellobiose) had a significant impact on MPP, while the alcohols and short-chain fatty acids (lactate, acetate, and propionate) did not (Table 1; Fig. 1). These naturally occurring substrates were added to stimulate general metabolism and to evaluate impacts on MPP and community diversity, with unamended incubations serving as a negative control. Addition of cellobiose decreased MPP ∼70% in NH slurries (*t* test, *P* = 0.0061), and ∼35% in HC slurries (*P* = 0.0029) relative to unamended controls. All other amendments had no significant impact on ambient MeHg concentrations relative to those in unamended controls. Analysis of variance (ANOVA) revealed that the background HC site overall fell into a separate grouping relative to the NOAA and NH sites with elevated THg and MeHg levels (Table 1). NH amended with lactate, ethanol, or methanol was distinctly grouped away from all others. Interestingly, these three treatments showed the highest MPP, suggesting that they were distinct from all other sites and site treatment combinations (Table 1). Further, while all other NOAA or NH treatment and treatment-site combinations grouped together, suggesting that they were not statistically different from one another, the cellobiose treatment in NOAA and NH grouped with the background HC, suggesting that the cellobiose amendment caused these samples to act as background due to the significant decrease in MPP (Table 1; Fig. 1).

**TABLE 1:** Ambient and spiked THg and MeHg concentrations and MPP results

Site	Amendment	Ambient conditions		Spiked conditions		MPP^*a*^		
		THg (μg/g)	MeHg (pg/g)	^201^THg (μg/g)	Me^201^Hg (pg/g)	μg MeHg/kg THg spiked/day (10^−4^)	%	Group^*b*^
HC	None	3.5 × 10^−3^ ± 0.3 × 10^−3^	1.1 ± 0.2	4.7 × 10^−3^ ± 0.1 × 10^−3^	0.7 ± 0.1	1.5 ± 0.2	0.015 ± 0.002	A
	Cellobiose	3.6 × 10^−3^ ± 0.2 × 10^−3^	0.7 ± 0.1	4.1 × 10^−3^ ± 0.5 × 10^−3^	0.4 ± 0.0	1.0 ± 0.1	0.010 ± 0.001	A
	Lactate	2.7 × 10^−3^ ± 0.2 × 10^−3^	1.1 ± 0.1	5.2 × 10^−3^ ± 0.2 × 10^−3^	0.8 ± 0.0	1.5 ± 0.1	0.015 ± 0.001	A
	Ethanol	2.7 × 10^−3^ ± 0.1 × 10^−3^	1.1 ± 0.2	4.5 × 10^−3^ ± 0.7 × 10^−3^	0.8 ± 0.2	1.8 ± 0.1	0.018 ± 0.001	AB
	Acetate	3.6 × 10^−3^ ± 0.7 × 10^−3^	0.9 ± 0.2	5.1 × 10^−3^ ± 0.2 × 10^−3^	0.6 ± 0.1	1.2 ± 0.2	0.012 ± 0.002	A
	Propionate	3.0 × 10^−3^ ± 0.3 × 10^−3^	1.0 ± 0.2	4.3 × 10^−3^ ± 0.5 × 10^−3^	0.5 ± 0.1	1.2 ± 0.1	0.011 ± 0.001	A
	Methanol	3.9 × 10^−3^ ± 0.3 × 10^−3^	0.9 ± 0.1	5.9 × 10^−3^ ± 0.5 × 10^−3^	0.6 ± 0.1	1.0 ± 0.2	0.010 ± 0.002	A
NOAA	None	1.2 ± 0.6	310 ± 11	4.7 ± 1.0	980 ± 240	2.1 ± 1.1	0.021 ± 0.011	AB
	Cellobiose	0.9 ± 0.3	330 ± 120	3.6 ± 0.4	950 ± 190	2.6 ± 0.6	0.026 ± 0.006	A
	Lactate	2.6 ± 2.8	450 ± 290	4.6 ± 0.3	1,300 ± 150	2.9 ± 0.3	0.029 ± 0.003	ABCD
	Ethanol	0.7 ± 0.3	230 ± 47	5.0 ± 1.4	1,600 ± 460	3.2 ± 1.7	0.032 ± 0.017	ABCD
	Acetate	1.2 ± 1.0	280 ± 150	5.1 ± 1.0	1,400 ± 530	2.7 ± 1.8	0.027 ± 0.018	ABC
	Propionate	0.6 ± 0.4	150 ± 52	4.4 ± 0.7	1,400 ± 480	3.2 ± 1.5	0.032 ± 0.015	ABCD
	Methanol	0.7 ± 0.2	210 ± 7	4.9 ± 1.1	1,400 ± 150	2.9 ± 0.9	0.028 ± 0.009	ABCD
NH	None	3.3 ± 0.7	2,700 ± 250	4.4 ± 0.5	2,000 ± 420	4.5 ± 0.7	0.045 ± 0.007	BCD
	Cellobiose	3.4 ± 1.2	1,600 ± 98	4.6 ± 0.3	640 ± 64	1.4 ± 0.1	0.014 ± 0.001	A
	Lactate	3.4 ± 0.7	1,900 ± 100	4.5 ± 0.7	2,400 ± 650	5.3 ± 0.8	0.053 ± 0.008	CD
	Ethanol	2.4 ± 0.2	1,900 ± 340	4.4 ± 0.4	2,400 ± 930	5.4 ± 2.0	0.054 ± 0.020	CD
	Acetate	3.2 ± 0.5	2,100 ± 330	4.8 ± 0.6	1,700 ± 330	3.5 ± 1.0	0.035 ± 0.010	ABCD
	Propionate	3.0 ± 0.8	1,600 ± 160	4.0 ± 0.6	1,400 ± 310	3.4 ± 0.4	0.034 ± 0.004	ABCD
	Methanol	4.3 ± 1.3	2,200 ± 150	3.9 ± 0.6	2,100 ± 290	5.5 ± 1.1	0.055 ± 0.011	D

a MPP, methylmercury production potential.

b In the "Group" column, different letters indicate a significant difference using Tukey’s honestly significant difference test and a family-wide error rate of 5%.

### Effects of carbon amendments on microbial community diversity.

**FIG 2:**
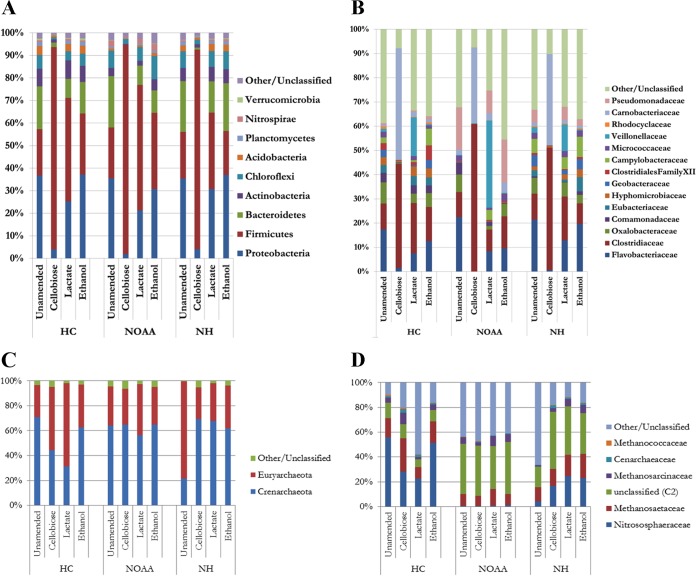
Relative abundances of bacterial phyla (A) or families (B) and of archaeal phyla (C) or families (D) detected for three sites (HC, NOAA, and NH) amended separately with cellobiose, lactate, or ethanol.

It was unknown at this point in the study whether the presence of cellobiose or a metabolic by-product thereof altered *hgcAB*^+^ microorganism abundance, if the amendment caused a preferential shift in the microbial community that did not favor *hgcAB*^+^ microorganisms, or if the C source enhanced demethylation (i.e., demethylators). To address this concern, the microbial community structure of select amendments was evaluated by 16S rRNA gene sequencing. Among the amendments for which sequencing was performed (unamended, cellobiose, lactate, and ethanol), as expected, cellobiose amendment had the largest impact on both the bacterial community structure (Fig. 2) and α-diversity (Table 2), mirroring the impacts of amendments on Hg methylation. Bacterial α-diversity was reduced by roughly 2/3 compared to that in the unamended incubations. The lactate and ethanol amendments yielded bacterial communities that largely mirrored the phylogeny of the *in situ*, untreated community (Fig. 2) and the α-diversity of the unamended slurries (Table 2) postincubation.

**TABLE 2:** 16S rRNA amplicon sequencing and α-diversity

Site	Carbon amendment	Bacteria		Archaea	
		No. of OTUs	Shannon diversity index	No. of OTUs	Shannon diversity index
HC	None	533	4.71	273	3.66
	Cellobiose	110	1.98	298	4.20
	Lactate	433	4.61	157	2.27
	Ethanol	459	4.71	233	3.83
NOAA	None	294	3.76	573	5.63
	Cellobiose	102	2.11	638	5.77
	Lactate	260	4.07	578	5.56
	Ethanol	344	3.93	607	5.65
NH	None	430	4.23	140	1.92
	Cellobiose	132	2.06	368	4.79
	Lactate	432	4.39	313	4.44
	Ethanol	468	4.28	346	4.59

Cellobiose amendments shifted the communities from all three sites strongly toward Firmicutes, although all of the amendments resulted in a higher fractional abundance of Firmicutes relative to that in the unamended controls. While the Firmicutes constituted ∼20% of the unamended communities, they constituted ∼35 to 50% of the bacterial community in ethanol- or lactate-amended slurries and ∼90% in cellobiose amendments (Fig. 2A). A closer inspection revealed that only three operational taxonomic units (OTUs), a Bacillus (*Trichococcus*) OTU and two Clostridium OTUs (Clostridium 1 and Clostridium 2) at relative proportions of ∼30 to 46%, ∼19 to 48%, and ∼11 to 22%, respectively, were predominant for all three sites. Using BLAST (40), *Trichococcus* matched best to *T. xienjiensis*, Clostridium 1 matched to *C. beijerinckii*, and Clostridium 2 matched to *C. mosignum* (see Table S4 in the supplemental material).

We expected that the carbon sources used here would have limited impact on archaeal diversity, as Archaea primarily utilize hydrogen, CO_2_, or other C_1_ compounds as carbon and electron sources (41, 42). In accordance with this expectation, archaeal α-diversity was similar among treatments at both NOAA and the background HC (Fig. 2C and D; Table 2), suggesting little effect. The downstream NH had the highest THg level, MeHg level, and MPP in this study (Table 1). In our previous work (31), NH showed the lowest native archaeal α-diversity, but here each carbon source, including cellobiose, increased the α-diversity to levels observed with the other sites and treatments. The effect of the carbon sources on particular methanogenic species varied with each site (Fig. 2C and D; see Table S5 in the supplemental material).

**FIG 3:**
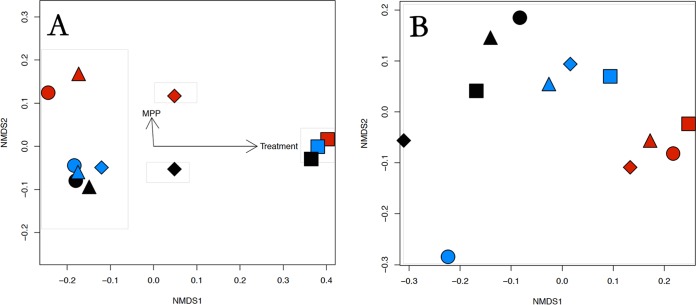
NMDS plots with ADONIS analysis for Bacteria (A) and Archaea (B) to determine the effect of carbon amendment on the β-diversity of the three sites used in this study. Black, Hinds Creek (HC) (the background site); red, NOAA (NO) (upstream); blue, New Horizon (NH) (downstream). Amendments: ●, unamended; ⬥, lactate; ▲, ethanol; ■, cellobiose.

While the α-diversity was substantially lower with cellobiose treatment for the Bacteria, the β-diversity also revealed that cellobiose treatment exerted a significant influence on the bacterial populations within the microbial communities for all three sites tested (Fig. 3A). The nonmetric multidimensional scaling (NMDS) plot in conjunction with ADONIS (Algorithm for Dynamic Optical Networks based on Internet Solutions) analysis revealed that the carbon amendment explained 77% of the variance in the bacterial community β-diversity (*P* = 0.002) and MPP explained an additional 10% of the variance (*P* = 0.004). As with the other analyses used in this study, the effect of carbon amendment on the β-diversity of the Archaea was highly variable and had no clear explanation other than that the NOAA samples appeared to be somewhat different than NH or the background HC (Fig. 3B).

### Effect of carbon amendments on the Hg-methylating community.

There are currently ∼140 microorganisms known to carry the Hg-methylating gene pair *hgcAB* within the Deltaproteobacteria, Firmicutes, and methanogenic Archaea (http://www.esd.ornl.gov/programs/rsfa/data.shtml). In the publicly available metagenomes, *hgcA* is seldom identified, and typically strains with a putative *hgcA* gene constitute <1% of the community populations (3). In this study, Hg-methylating organisms constituted up to 0.01% of all 16S rRNA gene sequences with sequence matches of >95% similarity (Table 3).

**TABLE 3:** 16S rRNA sequence matches to known Hg methylators

Species	Clade	No. of sequence matches											
		HC				NOAA				NH			
		Unamended	Amended with:			Unamended	Amended with:			Unamended	Amended with:		
			Cellobiose	Lactate	Ethanol		Cellobiose	Lactate	Ethanol		Cellobiose	Lactate	Ethanol
*Desulfobulbus propionicus*	Deltaproteobacteria	1	0	0	0	0	0	0	0	1	0	1	1
*Desulfomicrobium baculatum*	Deltaproteobacteria	0	0	0	0	0	0	0	0	1	0	0	0
*Geobacter bremensis*	Deltaproteobacteria	0	0	0	2	0	0	0	0	0	0	1	2
*Desulfomonile tiedjei*	Deltaproteobacteria	0	0	0	0	0	0	0	0	0	3	0	0
*Clostridium tunisiense*	Firmicutes	26	1	15	1	0	0	0	0	0	0	0	30
*Methanomethylovorans hollandica*	Archaea	25	16	7	15	1	0	0	0	8	7	4	28
*Methanomassiliicoccus luminyensis*	Archaea	38	24	4	4	5	4	6	2	3	8	5	10
*Methanolobus psychrophilus*	Archaea	5	12	1	4	0	0	0	0	4	0	4	3
*Methanocella paludicola*	Archaea	0	0	0	0	1	0	0	0	62	20	3	1
*Methanocella arvoryzae*	Archaea	1	14	4	13	28	30	58	24	2	1	3	2
*Methanoregula boonei*	Archaea	16	15	1	34	6	12	23	8	21	15	55	33
Methanospirillum hungatei	Archaea	0	1	0	2	0	0	0	0	0	1	2	2

Known bacterial Hg methylators, such as the family *Geobacteraceae*, were reduced to below detection from all incubations receiving cellobiose amendment (Fig. 2B; Table S4). These results suggest that the cellobiose treatment may have selected against Hg-methylating bacteria and resulted in both the community population shift and the lower MPP. Known Hg-methylating bacterial (i.e., *Geobacteraceae*) abundances were similar for ethanol amendment and the unamended samples for all sites, while lactate addition appeared to lower their populations slightly. Cellobiose was expected to stimulate the Firmicutes, since they are known to degrade lignocellulose and primary by-products such as cellobiose (37, 43). Overall, Firmicutes abundance increased with cellobiose addition (Fig. 2), but Hg-methylating Firmicutes were not among this group. Note that with respect to cellobiose, the lone *hgcAB*^+^ Clostridium sp. detected (*C. tunisiense*, a member of Clostridiales family XII) (Table 3) appeared to be inhibited by cellobiose at the background HC and below detection at NOAA and NH, except that ethanol was stimulatory at NH. The reasons for this effect are unclear, but it is plausible that *C. tunisiense* either degraded ethanol or utilized degradation products from sulfate-reducing bacteria, since the latter were reported to be stimulated by ethanol elsewhere (44). Members of the Euryarchaeota genus *Methanomicrobia*, the only known class of Archaea to methylate Hg (2), were in similar proportions postincubation at all sites (Fig. 2C and D; Table S5), with the exception of lactate treatment in the background HC site. However, their numbers were considerably lower than those of other families (Fig. 2D), as might be expected given the relative rarity of Hg methylators overall (3, 39). Given the lack of change in the MPP (Table 1), it is unlikely that Hg methylators were specifically stimulated by lactate, at least within the time frame of the carbon amendment incubation, 24 h. Interestingly, the downstream NH appeared to have a high proportion of *Methanomicrobia in situ*, but all three treatments reduced their numbers by ∼75% (Fig. 2). The reasons for this effect are unclear at this time.

With this method of comparison, it appears that the methanogens are the most abundant clade of Hg methylators throughout HC, NOAA, and NH. Few bacterial sequences identifying known Hg methylators were detected at HC and NH, and none were detected at NOAA (Table 3), although MeHg production was observed (Table 1). At the resolution of 16S rRNA gene sequencing, Hg methylator abundance (Table 3) did not correlate to changes in MeHg production (Table 1). Note that the low sequence variability between Hg-methylating and nonmethylating Firmicutes may make this analysis inappropriate for abundance estimations.

### Identification of Hg-methylating microorganisms with PCR-based methods.

**FIG 4:**
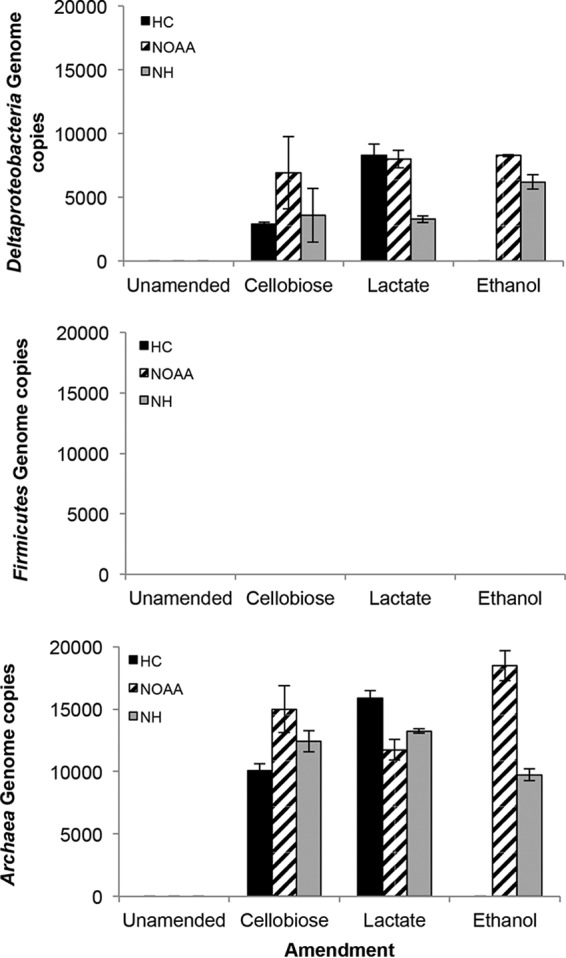
qPCR for *hgcA* specific to Deltaproteobacteria (top), Firmicutes (middle), and Archaea (bottom) at the three sites (HC, NOAA, and NH) for each of the treatments. *hgcA* quantification is shown. Detection limits (genome copies per 1 ng of gDNA template): Deltaproteobacteria, 2,000; Firmicutes, 200; and Archaea, 2,000 (empirically determined from a representative strain from each clade).

We recently designed qualitative *hgcAB* and quantitative *hgcA* clade-specific PCR-based methods to determine the presence and abundance of Deltaproteobacteria, Firmicutes, and methanogenic Archaea (11) (see Table S3 in the supplemental material). These protocols were developed to quantify the widest fraction of known Hg-methylating microorganisms, and to be as inclusive as possible, the primers ranged in degeneracy, from 8- to 512-fold, typically being <72-fold. We are not aware of any previous application of these protocols to diverse environmental samples. Hence, we used these protocols to further examine the presence (see Fig. S3 in the supplemental material) and abundance (Fig. 4; see Fig. S2 in the supplemental material) of Hg-methylating microorganisms. PCR for the 16S rRNA gene and *hgcAB* genes confirmed the presence of the latter with all carbon amendments except HC ethanol treatment and all unamended treatments, in which case it was below detection (Fig. S3). In those samples with detectable *hgcAB*, Deltaproteobacteria and Archaea *hgcA* genes were calculated at ∼3,000 to 8,500 and ∼10,000 to 16,000 genome copies per 1 ng of template genomic DNA (gDNA), respectively, with a detection limit of 2,000 genome copies (Fig. 4). Importantly, a single product was observed (by gel electrophoresis [Fig. S2] and melting curve analysis [data not shown]) from the amplified qPCR product for Deltaproteobacteria and Archaea for each sediment slurry experiment. Similar melting temperatures (*T_m_*) among the samples were observed, which we interpret to mean that the *hgcAB* genes being amplified have similar GC contents. One exception, the amplicon generated from gDNA isolated from HC amended with lactate, had a lower *T_m_* (∼82°C), while for all other samples the *T_m_* was ∼85°C. Assuming one gene per cell and that the strains amplified with similar efficiencies, Hg-methylating Archaea are 2 to 5 times more abundant at HC, NOAA, and NH than other Hg methylators, in agreement with the 16S rRNA gene count differences. With a variety of analyses, methanogens are proposed to be prevalent Hg methylators at a number of sites (9, 45). Our report describes the use of clade-specific *hgcA* qPCR primers to implicate methanogens as the most abundant methylators in environmental samples (11).

It is unclear why *hgcA* was not detected in some treatments given that net MeHg production occurred in these same treatments. Potentially, the Hg methylator relative abundance per nanogram of gDNA did not exceed the clade-specific and empirically determined detection limit, given their relative rarity. No product was detected for the Firmicutes *hgcA* qPCR, regardless of site or amendment, with a detection limit of 200 genome copies per reaction (11), even though the most abundant *hgcAB*^+^ bacterial OTU was *C. tunisiense*. Examination of the putative Clostridium sp. *hgcAB* sequences against the Firmicutes *hgcA* primers, including *C. tunisiense*, revealed typically >5 mismatches. This high number of mismatches may account for the inability to identify the *hgcA* from *C. tunisiense*, the most abundant OTU. The *Firmicutes hgcA* qPCR primers (11) were specific to sulfate- and sulfite-reducing Firmicutes and not the fermentative ones and therefore would not be expected to amplify *C. tunisiense*. To determine whether we could quantify *hgcA* in *C. tunisiense*, we designed species-specific primers for *hgcA* in this organism. Results for all samples were below detection at the standard 30 cycles, although correctly sized products were observed after 37 cycles (data not shown). As detailed in our earlier work (11), 40 cycles can be used, but this usually results in nonspecific amplification of other organisms both within and outside the selected clade and so is not recommended.

### Conclusions.

The goals of this study were to determine the relative abundance of each Hg-methylating clade at the East Fork Poplar Creek site and to ascertain the effects of various carbon and electron sources on net Hg methylation. Detection and quantification of *hgcAB* within cells as well as at the microbial community level is challenging for several reasons. In individual species, the genes are not essential, as demonstrated by gene deletion experiments in which the Δ*hgcAB* mutants survive, but they are putatively involved in central carbon metabolism (39). If so, the genes are expected be expressed at a low and constitutive level, as recently demonstrated by transcriptional analysis of cultures with different electron donors and acceptors in *Desulfovibrio dechloracetivorans* BerOc1 (46). At the community level, the same challenges exist for systems biology efforts such as transcriptomics and proteomics. However, the relative rarity of organisms that possess *hgcAB*, apparently occurring only in anaerobes, further extends the difficulty of detection and quantification. These factors increase the difficulty of determining how environmental parameters such as geochemistry and nutrient availability influence viability of Hg methylators as well as *hgcAB* expression and HgcAB protein function. Using different carbon and electron sources, in this study we have been able to show through a variety of analyses that the methanogenic Archaea are likely the primary Hg-methylating organisms in these samples. Perhaps more importantly, while common anaerobic metabolites such as lactate and alcohols may increase Hg methylation to some degree the apparent excess of cellobiose, a lignocellulosic degradation by-product, significantly inhibited Hg methylation. Further work may reveal relatively simple strategies to reduce MeHg generation and act to inform site risk management and future remediation strategies.

## MATERIALS AND METHODS

### Sediment sampling and initial analysis.

Sediment samples were collected from the stream bottom at two locations in EFPC and one in Hinds Creek, an uncontaminated stream with similar geochemistry and hydrology (see Fig. S1 in the supplemental material). EFPC originates in and runs beside the Y-12 facility and has historically received Hg-containing discharges through both surface and subsurface hydrological seepage. The HC site acted as a control for the planned experiments. The two EFPC sites, here referred to as NOAA (designation EFK22.3; 36°0′8.40″N and 84°14′56.10″W) and NH (designation EFK5; 35°57′46.56″N and 84°21′33.00″W), are 4 km and 21 km downstream of the Y-12 point source, respectively (27, 33). Three cores were collected for each site by hand with polycarbonate core barrels (4.5 cm [inner diameter] by 30 cm) and placed on ice for return to the laboratory within 1 h. Once back in the laboratory each core was immediately sectioned in an anaerobic chamber (Coy Laboratory Products, Inc., Grass Lake, MI) (95% N_2_, 5% H_2_) by depth (0 to 4 cm, 4 to 8 cm, and 8 to 12 cm), and a composite sample was created by combining the sediments at each depth from the three cores. A portion of the composite sediment for each depth interval was used for initial testing of ambient THg and MeHg concentrations (as described below) to determine the depth with the highest MeHg concentration, to make preliminary measurements of MPP as a function of depth, and to determine the needed amounts of ^201^Hg to add to sediments for the MPP experiments. The remaining composite sample was stored at 4°C for ∼3 days for the carbon amendment experiments while the initial assessments were conducted.

### Assessment of MPP in sediment cores with depth.

The MeHg production potential (MPP) by microbial communities is also impacted by geochemistry (e.g., pH) (19), hydrology (47), Hg and MeHg concentrations and bioavailability, and net accumulation of MeHg (48). MPP is the amount of MeHg produced per day (micrograms of MeHg per kilogram of THg spiked per day). The MPP in sediment depth sections was estimated with enriched stable isotope spikes (49), ^201^HgCl_2_ (Oak Ridge National Laboratory [ORNL]; 98.1% purity). Target spike levels were similar to ambient Hg concentrations (see Table S1 in the supplemental material). Assays were conducted in triplicate by mixing 5 g of fresh wet sediment with 5 ml of filter-sterilized (0.2 μm) and degassed river water from the respective sites in sterile glass Balch-Hungate tubes within the anaerobic chamber. Tubes were capped with a Teflon stopper and aluminum crimp tops to maintain anaerobic conditions. ^201^Hg was injected to start the assay. Tubes were incubated in the dark at 25°C for 24 h. At the end of the incubation, assays were stopped by freezing at −20°C. Ambient and enriched isotope concentrations of THg and MeHg were then determined for each sample. The MPP is a rate constant, calculated as the fraction of the ^201^Hg spike converted to Me^201^Hg in 24 h. Since ambient concentrations were considerably increased and since newly introduced Hg is more bioavailable for Hg(II) methylation than ambient Hg, the assay provides a potential rate constant for comparison among sites and enrichments. MPPs are given as the average from triplicate samples with standard deviation.

### Carbon substrate amendment.

To examine the influence of carbon amendments on Hg methylation, sediment slurries made from composite 8- to 12-cm-depth sediments from each site were amended separately with a variety of organic substrates, and MPP was evaluated simultaneously as described above. Each set of triplicate tubes was amended separately with 50 mM each substrate, which included cellobiose, lactate, ethanol, methanol, propionate, or acetate, while one set was not amended and acted as the control. Each sample was mixed thoroughly (i.e., slurry) and immediately spiked with inorganic ^201^Hg isotope as per the initial experiment. All other conditions were the same as described for the initial incubations. After 24 h, samples were divided and stored at −20°C prior to Hg isotope determination (as described above), and community genomic DNA (gDNA) extraction was followed by pyrosequencing and quantification of *hgcA* genome copy numbers (as described below). Only the unamended incubations and those amended with cellobiose, lactate, or ethanol were sequenced, as these conditions appeared to have an effect on MeHg production as described below. To evaluate differences in MPP among enrichments, we employed an ANOVA followed by *post hoc* Welch's unequal-variance *t* tests (50) comparing the unamended samples with each of the individual carbon amendments within each of the three sites, with the Holm-Bonferroni method of correcting for multiple comparisons (51).

### THg and MeHg determination.

THg and MeHg concentrations were determined by a modification of EPA methods 1630 and 1631. Details of the methods are described elsewhere (49, 52), and only an overview of this method is provided here. Sediments for both THg and MeHg determination were extracted with a mixture of potassium bromide, sulfuric acid, and copper sulfate (52, 53). After this initial extraction, the sample was split evenly for THg and MeHg analyses. The sample for THg analysis was further digested with *aqua regia*. The aliquot for MeHg analysis was extracted with methylene chloride. Analyses for ambient and isotopic THg and MeHg concentrations were conducted with a purge-and-trap system (Brooks Rand Merx) coupled with an inductively coupled mass spectrometer (ICP-MS) (PerkinElmer Elan DRC) (31, 49).

### DNA extraction and pyrosequencing of the SSU rRNA gene.

For each sample, 3 g of frozen sediment was used for gDNA extraction by the ORNL method (54, 55). Total gDNA concentrations and purity as assessed by *A*_260_/*A*_280_ and *A*_260_/*A*_230_ ratios were determined with Qubit (Thermo Fisher Scientific, Waltham, MA) and NanoDrop ND-1000 (Thermo Fisher Scientific), respectively (see Table S2 in the supplemental material). For 16S rRNA small-subunit (SSU) gene sequencing, oligonucleotides were purchased from Integrated DNA Technologies (IDT) (Coralville, IA). Separate sets of primers targeting the V1 to -3 hypervariable regions of the SSU rRNAs of Bacteria and Archaea were used for PCR amplification of the respective 16S rRNA genes (35). For Bacteria, the primers used were 27YMF (AGAGTTTGATYMTGGCTCAG) and 534R (TYACCGCGGCTGCTGG) to obtain an approximate amplicon length of 431 to 550 bp. For Archaea, the primers used were A2FA (TCYSGTTGATCCYGCSRG) and 571R (GCTACRGVYSCTTTARRC) for an approximate amplicon length of 479 to 1,221 bp. These primer sets were designed with GS FLX titanium paired-end adapters (Roche, Branchburg, NJ) and 8- to 10-nucleotide (nt) barcodes for sample multiplexing. Each PCR was set up in a 50-μl PCR mix with high-fidelity AccuPrime *Pfx* DNA polymerase (Invitrogen, Carlsbad, CA) following previously established protocols (33). Each PCR amplicon was purified with the Agencourt AMPure solid-phase paramagnetic bead technology (Agencourt Bioscience Corporation, Beverly, MA) following the manufacturer's protocol, and the concentration and size were estimated with an Agilent 2100 Bioanalyzer (Agilent Technologies, Inc., Waldbronn, Germany) with DNA 1000 reagents. The reaction products were pooled according to DNA quantity and quality prior to performing emulsion reactions for sequencing on a 454 Life Sciences Genome Sequencer FLX (Roche Diagnostics, Indianapolis, IN) with the unidirectional amplicon library sequencing protocol with an emPCR kit II and FLX titanium chemistry (Roche). The 16S rRNA gene sequences are available from MG-RAST with the accession numbers mgm4758641.3 to mgm4758688.3.

### 16S rRNA amplicon sequence analysis.

The 16S rRNA amplicon sequences were analyzed in MOTHUR (v. 1.26) (56), QIIME (v. 1.5.0) (57), and AmpliconNoise (v.1.24) (58). All samples were passed through the AmpliconNoise pipeline to remove both sequencing and PCR errors and chimeras with its Perseus algorithm and trimmed to 250 bp. Sequences were aligned in MOTHUR against the sequences from the Ribosomal Database Project (RDP) (release 10) (59) for Bacteria and Archaea and trimmed to preserve an approximate average length of 400 nt. Pairwise distances were calculated in MOTHUR and then clustered based on average linkage clustering. The OTUs were defined at a 97% similarity cutoff for all of our analyses. A BIOM format OTU table was implemented in the QIIME pipeline for diversity analysis, classification, and assigning taxonomy to the OTUs. The taxonomy reference databases from RDP were used for assigning taxonomy to Bacteria and Archaea OTUs. In order to identify the presence of the relatively rare 16S rRNA gene sequences of *hgcA*-containing microorganisms, all unique sequences, including singletons, were catalogued. BLAST (40) was used on each 16S rRNA gene sequence against cultured isolates confirmed to have *hgcA*. All hits with at least 150 bp of alignment and greater than 95% similarity were kept, with the presumption that 97% would be the same species. The Shannon diversity index (α-diversity) was calculated by first removing singletons (i.e., OTUs with only one read) and rarefied so that all samples had the same number of sequence reads, 2,194 for Bacteria and 2,716 for Archaea. Pairwise-abundance-weighted UniFrac distances between samples for the OTU table were used to create nonmetric multidimensional scaling plots with the vegan library (60) within the R software package (61). To accomplish this, MUSCLE (62) was used for the sequence alignments and Fasttree (63) was used for tree construction to enable UniFrac calculations, both using standard settings.

While α-diversity reveals the microbial community diversity within each site, β-diversity determines the differences in microbial community makeup between sites. For ordinating the β-diversity between the three sites and three representative carbon source treatments (cellobiose, lactate, and ethanol) used in this study, we employed nonmetric multidimensional scaling (NMDS). In conjunction with NMDS analysis, we also used ADONIS (Algorithm for Dynamic Optical Networks based on Internet Solutions) to determine the effect that each of the three carbon sources had on microbial community β-diversity. ADONIS is essentially a permutational nonparametric multivariate ANOVA (MANOVA) (64, 65).

### Analysis of *hgcAB* by PCR or of *hgcA* by qPCR.

The presence of *hgcAB*^+^ microbes was assessed using our recently developed universal degenerate PCR primer set (ORNL-HgcAB-uni-F [5′-AAYGTCTGGTGYGCNGCVGG-3′] and ORNL-HgcAB-uni-R [5′-CABGCNCCRCAYTCCATRCA-3′]) (11). These primers amplify a region that spans the adjacent *hgcA* and *hgcB* genes, both of which are necessary for Hg methylation in microorganisms (39). This primer set was specifically developed to amplify the widest possible fraction of known Hg-methylating organisms by testing against several dozen species across all three clades of methylators (11). Oligonucleotides were purchased from Integrated DNA Technologies (IDT) (Coralville, IA). For determining the presence of Hg methylators in all incubations, the broad-range *hgcAB* amplification protocol was followed (11) (see Table S3 in the supplemental material). Each PCR was set up with 10 ng of gDNA template in a 20-μl reaction mix with Platinum *Taq* DNA polymerase (Thermo Fisher Scientific) and processed on a Mastercycler Pro (Eppendorf, Hauppauge, NY). PCR products were visualized in agarose gels.

The abundance of microbes in each of the three dominant clades of methylators (Deltaproteobacteria, Firmicutes, and methanogenic Archaea) was measured relative to a known standard for each clade with our clade-specific degenerate *hgcA* qPCR primer (11) (Table S3). The primers were as follows: for Deltaproteobacteria, ORNL-Delta-HgcA-F (5′-GCCAACTACAAGMTGASCTWC-3′) and ORNL-Delta-HgcA-R (5′-CCSGCNGCRCACCAGACRTT-3′); for Firmicutes, ORNL-SRB-Firm-HgcA-F (5′-TGGDCCGGTDARAGCWAARGATA-3′) and ORNL-SRB-Firm-HgcA-R (5′-AAAAGAGHAYBCCAAAAATCA-3′); and for Archaea, ORNL-Archaea-HgcA-F (5′-AAYTAYWCNCTSAGYTTYGAYGC-3′) and ORNL-Archaea-HgcA-R (5′-TCDGTCCCRAABGTSCCYTT-3′). Like our universal primers, the clade-specific qPCR primers were specifically developed to amplify the widest possible fraction of known Hg-methylating organisms in each clade by testing against many species. The qPCR protocols for all three primer sets have been reported previously (11) (Table S3). Each qPCR was set up with 5 ng of template in a 20-μl reaction mix with iQ SYBR Green Supermix (Bio-Rad, Hercules, CA) and processed on a C1000 Touch real-time PCR detection system (Bio-Rad). Template concentrations were determined by Qubit (Thermo Fisher Scientific) (Table S2). Data were analyzed with CFX Manager (version 3.1; Bio-Rad) by single-threshold analysis (66, 67). Additional analysis was required for samples processed by the Archaea protocol with an Agilent 2100 Bioanalyzer (Agilent Technologies, Inc.) to separate the expected peak (125 bp) from a nonspecific product (∼50 bp). For each clade-specific primer set, a standard curve (six 10-fold serial dilutions, 1 × 10^7^ to 1 × 10^1^ gDNA copies per reaction) was performed to calculate primer efficiency and determine genome copy number for each sample. The qPCR standard curves were developed with gDNA from pure *hgcAB^+^* cultures, specifically, Desulfovibrio desulfuricans ND132 (for Deltaproteobacteria), *Desulfitobacterium metallireducens* (for Firmicutes), and *Methanomethylovorans hollandica* (for Archaea).

For *Clostridium tunisiense*, species-specific *hgcA* gene primers were designed and were located at the same gene locus as the Firmicutes clade-specific qPCR primers (11). The *C. tunisiense* primers were designated ORNL-F-C661DRAFT-F (5′-TGGGCCAATAAGAAGCGAGGATA-3′) and ORNL-F-C661DRAFT-R (5′-GTAGAATTATTACTAAAGCTA-3′). All protocols were identical to those used for the Firmicutes clade qPCR primers as described above.

## Supplementary Material

Supplemental material
