# Supplementary material for: Carbon Amendments Alter Microbial Community Structure and Net Mercury Methylation Potential in Sediments
Source: Appl Environ Microbiol. 2018 Jan 17;84(3):e01049-17. doi: 10.1128/AEM.01049-17 (PMC5772229; doi:10.1128/AEM.01049-17)
Supplement: Supplemental material [file supp_84_3_e01049-17__index.html]

Supplemental material 

# Carbon Amendments Alter Microbial Community Structure and Net Mercury Methylation Potential in Sediments

## Supplemental material

- Supplemental file 1 -

  Supplemental methods; map/aerial images (Fig. S1); gel electrophoresis (Fig. S2); PCR (Fig. S3).

  PDF, 717K
- Supplemental file 2 -

  Four-centimeter-depth interval measurements among environmental sites (Table S1); DNA concentration prior to pyrosequencing and qPCR (Table S2); amplification protocols (Table S3); 16S bacterial sequencing (Table S4); archaeal sequencing (Table S5).

  XLSX, 616K
