# Supplementary material for: Carbon Amendments Alter Microbial Community Structure and Net Mercury Methylation Potential in Sediments
Source: Appl Environ Microbiol. 2018 Jan 17;84(3):e01049-17. doi: 10.1128/AEM.01049-17 (PMC5772229; doi:10.1128/AEM.01049-17)
Supplement: Supplemental material [file AEM.01049-17_zam003188273s1.pdf]

Supplemental Methods Text
The following coding was used in R to determine the β-diversity for Bacteria and *Archaea* in this study.

Bacteria

Call:
adonis(formula = umat ~ metdat$Treatment + metdat$MPP, strata = metdat$Site)

Blocks: strata
Permutation: free
Number of permutations: 999

Terms added sequentially (first to last)

	Df	SumsOfSqs	MeanSqs	F.Model	R2	Pr(>F)
metdat$Treatment	3	0.72179	0.240596	13.7688	0.76849	0.002 **
metdat$MPP	1	0.09512	0.095119	5.4435	0.10127	0.004 **
Residuals	7	0.12232	0.017474	0.13023		
Total	11	0.93922	1.00000			

Signif. codes: 0 '***' 0.001 '**' 0.01 '*' 0.05 '.' 0.1 ' ' 1

Archaea

Call:
adonis(formula = umat ~ metdat$Treatment + metdat$MPP, strata = metdat$Site)

Blocks: strata
Permutation: free
Number of permutations: 999

Terms added sequentially (first to last)

	Df	SumsOfSqs	MeanSqs	F.Model	R2	Pr(>F)
metdat$Treatment	3	0.08631	0.028769	0.4804	0.11734	0.665
metdat$MPP	1	0.23000	0.230005	3.8408	0.31272	0.603
Residuals	7	0.41920	0.059885	0.56994		
Total	11	0.73551	1.00000			

Supplemental Figures:

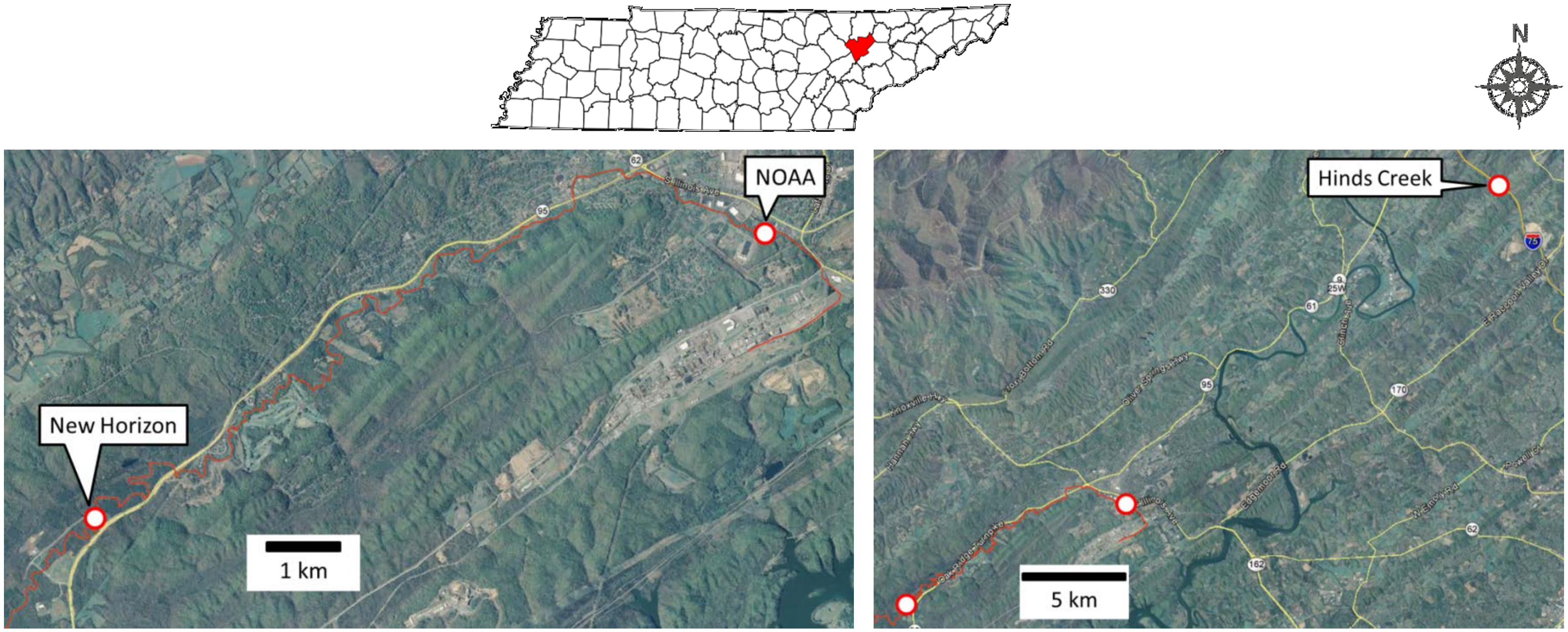

Figure S1: Sediment cores were isolated from three locations within Anderson County, TN (highlighted in red). (Left) Samples were taken from two locations from the mercury contaminated East Fork Poplar Creek (EFPC) including at the New Horizon (NH) and NOAA sites. (Right) Hinds Creek (HC), approximately 20 km northeast of EFPC, was selected as a background site with minimal THg and MeHg concentrations. Aerial views were acquired using Google Earth.

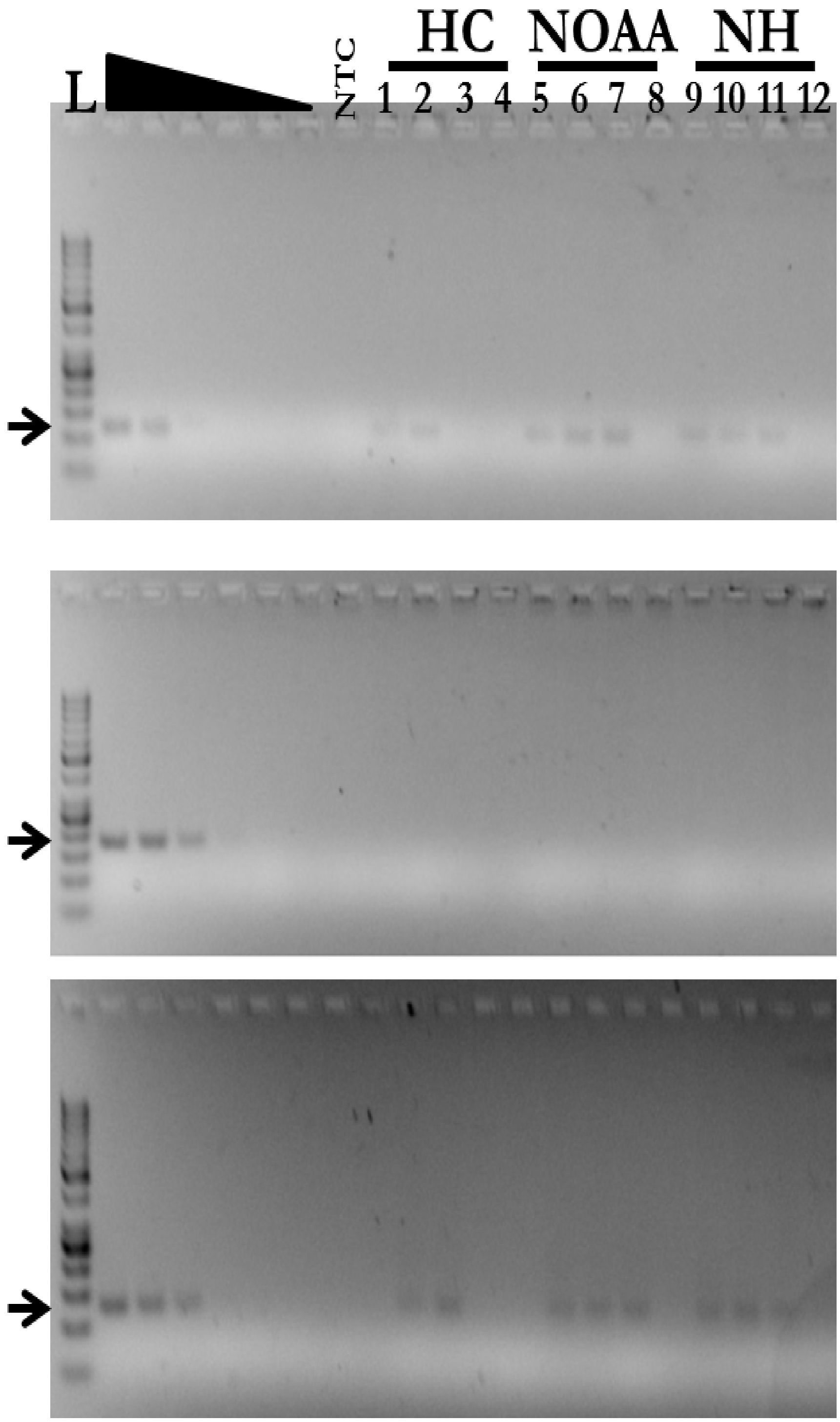

Figure S2: Gel electrophoresis. Cellobiose (1, 5, 9), Lactate (2, 6, 10), Ethanol (3, 7, 11) and Unamended (4, 8, 12). Ladder (L) is 50 bp from New England BioLabs. Arrow denotes expected product size specific to *Deltaproteobacteria* (107 bp), *Firmicutes* (167 bp) and *Archaea* (125 bp). The black triangle represents decreasing concentration of the standard curve (serial 10-fold dilution, 1 x 10^6 - 1 x 10^1) *of Desulfovibrio desulfuricans* ND132 (*Deltaproteobacteria*), *Desulfitobacterium metallireducens* (*Firmicutes*), and *Methanomethylovorans hollandica* (*Archaea*), respectively.

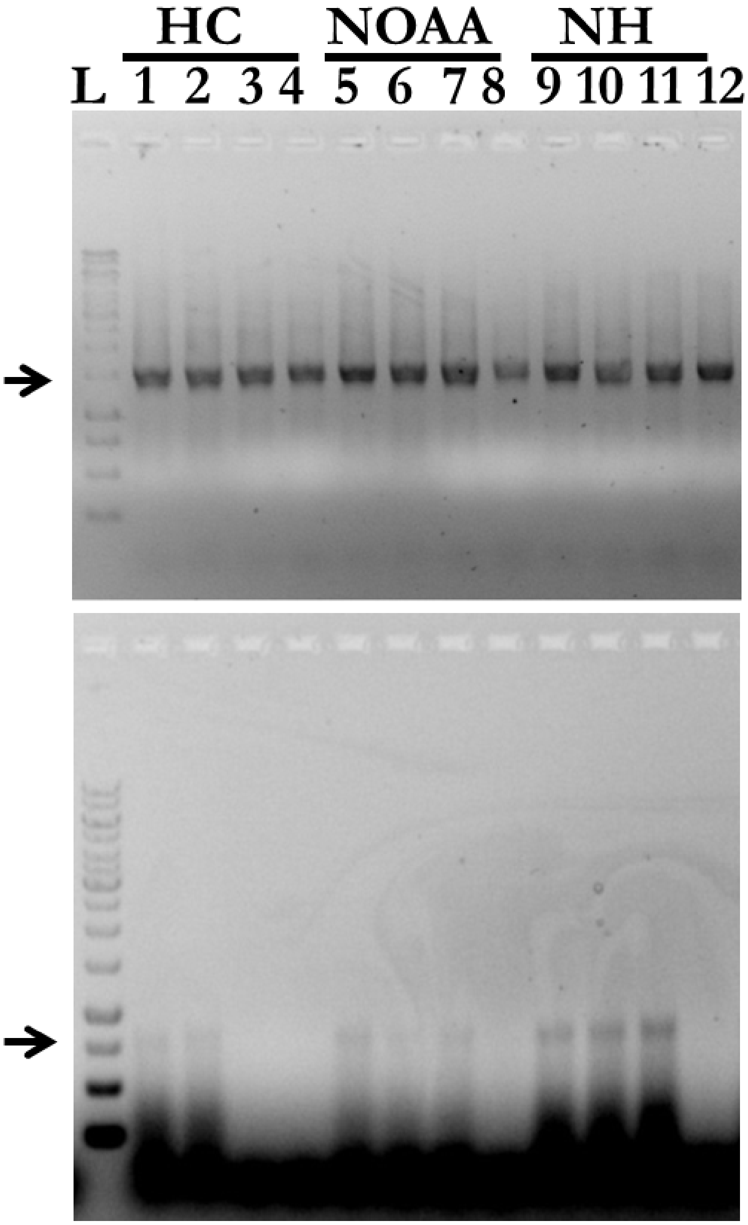

Figure S3: PCR for 16S rRNA gene (Top) and *hgcAB* (Bottom) of the three sites (HC, NOAA, and NH) for each of the treatments: Cellobiose (1, 5, 9), Lactate (2, 6, 10), Ethanol (3, 7, 11) and Unamended (4, 8, 12). Ladder (L) is 1kb Plus from Thermo Fisher Scientific. Arrow denotes expected product size for 16S (~1500 bp) and *hcgAB* (~750-1100 bp).
